# Multivariate network meta-analysis of pharmacological interventions for the treatment of acute bipolar mania: a bayesian approach using lognormal prior distribution

**DOI:** 10.1192/j.eurpsy.2023.1208

**Published:** 2023-07-19

**Authors:** P. K. Malo, B. Bhaskarapillai, M. Kesavan

**Affiliations:** 1 Centre for Brain Research, Indian Institute of Science; 2 Department of Biostatistics; 3 Department of Psychiatry, National Institute of Mental Health & Neuro Sciences, Bengaluru, India

## Abstract

**Introduction:**

Conventional Bayesian network meta-analysis (NMA) of multiple outcomes are performed using non-informative prior distribution, independently for each outcome.

**Objectives:**

This study aimed to estimate pharmacological intervention effects against placebo within a multivariate Bayesian framework using an informative lognormal prior distribution.

**Methods:**

13,188 participants were evaluated for two dichotomous study outcomes, namely, treatment response and all-cause dropouts, in 57 double-blinded randomized controlled trials (RCTs) for the treatment of acute bipolar mania (ABM) in adults. Both the study outcomes were measured from baseline to week 3. 10 pharmacological drugs or interventions consisted of mood stabilizers, anti-psychotics, antidepressants, combinations of the above and other agents, and were compared against each other as well as with placebo either as monotherapy or add on agents. These treatments include placebo, aripiprazole, haloperidol, quetiapine, ziprasidone, olanzapine, divalproex, paliperidone, carbamazepine, lithium; and lamotrigine. Aggregated arm-based data on both the study outcomes were considered. We used the *logit* scale to model the probability of event occurrence and adopted multivariate modelling approach; wherein both the study outcomes were included in a single NMA model. Further, the between-study variance-covariance matrix was decomposed using the Cholesky and spherical decomposition techniques and the results were compared. The deviance information criterion (DIC) indices were used to assess the model fit. Analyses included 16,00,000 Markov Chain Monte Carlo (MCMC) iterations with 6,00,000 burn-in period and thinning of 100; tested by running three chains with different starting values. All the analyses were carried out in WinBUGS software.

**Results:**

Under Cholesky and spherical decompositions, the correlation between the study outcomes were estimated as -0.51 (-0.68, -0.29) and -0.56 (-0.68, -0.50), respectively. DIC model fit index values for Cholesky and spherical decompositions were 667.74 and 667.53, respectively; indicating both decomposition techniques were equally good. Further, the Gelman-Rubin convergence statistics were stable and all Monte Carlo errors were around 0.005. Overall, olanzapine, paliperidone and quetiapine were both significantly more effective and acceptable than placebo; whereas aripiprazole, haloperidol ziprasidone, divalproex, and carbamazepine were not. In addition, both lithium and lamotrigine failed to be effective and acceptable.

**Conclusions:**

Our findings exhibit an excellent concordance with the one used in clinical practice. Moreover, the Canadian Network for mood and Anxiety Treatments, and Royal Australian and New Zealand College of Psychiatrists guidelines also recommended these drugs as first-line medications for treating bipolar disorder.

**Disclosure of Interest:**

None Declared

